# Skin toxicity and quality of life in patients with metastatic colorectal cancer during first-line panitumumab plus FOLFIRI treatment in a single-arm phase II study

**DOI:** 10.1186/1471-2407-12-438

**Published:** 2012-09-29

**Authors:** Josef Thaler, Meinolf Karthaus, Laurent Mineur, Richard Greil, Henry Letocha, Ralf Hofheinz, Eva Fernebro, Erick Gamelin, Ana Baños, Claus-Henning Köhne

**Affiliations:** 1 Department of Internal Medicine IV - Haematology and Oncology, Hospital Wels-Grieskirchen, Wels, Austria; 2 Department of Haematology and Oncology, Neuperlach Hospital, Munich, Germany; 3 Department of Oncology and Radiotherapy, Institute Sainte-Catherine, Avignon, France; 4 IIIrd Medical Department with Hematology and Medical Oncology, Oncologic Center, Paracelsus Medical University Salzburg, Salzburg, Austria; 5 Oncology Clinic, Västerås Hospital, Västerås, Sweden; 6 Day treatment centre at the Interdisciplinary Tumour Centre Mannheim, University Medical Centre, Mannheim, Germany; 7 Department of Oncology, Lund University Hospital, Lund, Sweden; 8 Department of Oncology, Paul Papin Cancer Institute, Angers, France; 9 Department of Biostatistics, Amgen Limited, Uxbridge, UK; 10 Department of Oncology and Haematology, Oldenburg Hospital, Oldenburg, Germany

**Keywords:** Colorectal cancer, Panitumumab, Quality of life, Tolerability

## Abstract

**Background:**

Integument-related toxicities are common during epidermal growth factor receptor (EGFR)-targeted therapy. Panitumumab is a fully human monoclonal antibody targeting the EGFR that significantly improves progression-free survival when added to chemotherapy in patients with metastatic colorectal cancer who have wild-type (WT) *KRAS* tumours. Primary efficacy and tolerability results from a phase II single-arm study of first-line panitumumab plus FOLFIRI in patients with metastatic colorectal cancer have been reported. Here we report additional descriptive tolerability and quality of life data from this trial.

**Methods:**

Integument-related toxicities and quality of life were analysed; toxicities were graded using modified National Cancer Institute Common Toxicity Criteria. Kaplan-Meier estimates of time to and duration of first integument-related toxicity were prepared. Quality of life was measured using EuroQoL EQ-5D and EORTC QLQ-C30. Best overall response was analysed by skin toxicity grade and baseline quality of life. Change in quality of life was analysed by skin toxicity severity.

**Results:**

154 patients were enrolled (WT *KRAS* n = 86; mutant *KRAS* n = 59); most (98%) experienced integument-related toxicities (most commonly rash [42%], dry skin [40%] and acne [36%]). Median time to first integument-related toxicity was 8 days; median duration was 334 days. Overall, proportionally more patients with grade 2+ skin toxicity responded (56%) compared with those with grade 0/1 (29%). Mean overall EQ-5D health state index scores (0.81 *vs.* 0.78), health rating scores (72.5 *vs.* 71.0) and QLQ-C30 global health status scores (65.8 *vs.* 66.7) were comparable at baseline *vs.* safety follow-up (8 weeks after completion), respectively and appeared unaffected by skin toxicity severity.

**Conclusions:**

First-line panitumumab plus FOLFIRI has acceptable tolerability and appears to have little impact on quality of life, despite the high incidence of integument-related toxicity.

**Trial registration:**

ClinicalTrials.gov NCT00508404

## Background

Many agents are now available for the treatment of metastatic colorectal cancer, and combining 5-fluorouracil (5-FU)-based chemotherapy with novel targeted agents is commonplace. FOLFIRI (folinic acid, infusional 5-FU and irinotecan) is frequently used in the first- and second-line settings [1], where it is often given in combination with the vascular endothelial growth factor A-targeted agent, bevacizumab [2]. Addition of epidermal growth factor receptor (EGFR)-targeted agents (e.g. the chimeric antibody cetuximab or the fully human antibody panitumumab) to FOLFIRI also significantly improves outcomes in patients with wild-type (WT) *KRAS* tumours [3,4]. As median overall survival (OS) in patients with metastatic colorectal cancer now approaches 2 years [5], long-term tolerability of treatment is important.

Although patients have undoubtedly benefitted from the integration of novel agents, the use of combination regimens inevitably leads to greater treatment-related toxicity [6] and potentially, adverse effects on quality of life. While FOLFIRI is associated with severe diarrhoea and neutropenia [7], EGFR inhibitors are associated with unique side effects, mostly different to those seen during chemotherapy. Because of the key role of EGFR signalling in skin [8], anti-EGFR therapy frequently results in integument-related toxicities [9]. Acneiform rash is the most common but xerosis, paronychia and trichomegaly also occur [10]. Although dermatological toxicities often resolve upon therapy discontinuation [11], these side effects can negatively affect treatment compliance and quality of life [12]. They can also lead to dose modification or discontinuation [13], thus potentially affecting the overall effectiveness of anti-EGFR treatment. Maintenance of quality of life is therefore an important treatment goal and patient-reported outcomes (PROs) are a useful way of measuring the impact of treatment on quality of life.

Study 314 (NCT00508404) was a single-arm, multicentre, phase II study evaluating the efficacy and safety of panitumumab plus FOLFIRI as first-line treatment for patients with metastatic colorectal cancer. In this study, consistently favourable efficacy was observed in patients with WT *vs.* mutant (MT) *KRAS* tumours [14]. Here we report additional descriptive tolerability and quality of life data from Study 314.

## Methods

### Study design and treatments

At the time of study initiation, the value of tumour *KRAS* status in patients receiving anti-EGFR therapies was unknown but after its importance was demonstrated [15,16] the protocol was amended to evaluate outcomes by *KRAS* status (the study was fully enrolled at this time). Panitumumab and FOLFIRI were administered once every 14 days until disease progression, unacceptable toxicity, or consent withdrawal. If FOLFIRI or panitumumab were withdrawn/withheld due to toxicity, the other agent could be continued. On Day 1 of the first cycle, panitumumab (6 mg/kg) was administered as a 60 ± 15 min intravenous (IV) infusion, just prior to chemotherapy; if well tolerated, subsequent infusions could be administered over 30 ± 10 min. No panitumumab-specific premedication was required. FOLFIRI (irinotecan [180 mg/m^2^ IV over 90 ± 15 min and leucovorin [400 mg/m^2^ IV over 120 ± 15 min [given sequentially/in parallel], followed by a 5-FU 400 mg/m^2^ bolus and a 5-FU 2400–3000 mg/m^2^ continuous IV infusion over 46 ± 2 h) was also administered on Day 1 of each cycle. One cycle was defined as the 14-day period following treatment initiation. The study protocol was approved by the relevant independent ethics committees and the study was conducted in accordance with International Conference on Harmonization of Good Clinical Practice regulations/guidelines.

### Patients

Eligible patients were ≥18 years of age, with histologically/cytologically confirmed, radiologically measurable metastatic colorectal cancer, and an Eastern Cooperative Oncology Group (ECOG) performance status of 0–2. All disease sites must have been evaluated ≤28 days prior to enrolment and tissue from the primary/metastatic site had to be available. Patients who had received prior systemic metastatic colorectal cancer therapy (including anti-EGFR therapy) were excluded; adjuvant fluoropyrimidine-based chemotherapy ≥6 months prior to enrolment was permitted. Radiotherapy ≤14 days prior to enrolment was not allowed and patients must have recovered from all radiotherapy-related toxicities. Patients with untreated and symptomatic central nervous system metastases or significant cardiovascular disease were excluded. All patients provided signed, informed consent.

### KRAS analyses

DNA was extracted from pre-treatment tumour samples to evaluate the *KRAS* mutation status and define the primary analysis population. *KRAS* testing was performed centrally at HistoGeneX in Belgium using the DxS kit that utilises allele-specific, real-time polymerase chain reaction to detect seven of the most common *KRAS* mutations.

### Integument-related toxicity analyses

The incidence and severity of adverse events (AEs) were measured continually throughout the study and graded using National Cancer Institute Common Toxicity Criteria (NCI CTC v3.0) [17]. Selected skin toxicities (nail changes, erythema, pruritus, acneiform rash, rash/desquamation, and ulceration) were graded using a modified version of these criteria [18]. A safety follow-up visit was scheduled for 8 weeks after treatment completion. To account for any potential imbalance in exposure between *KRAS* groups, patient incidence rates for exposure-adjusted AEs were analysed. The exposure-adjusted patient incidence was calculated as the number of patients experiencing the event divided by the sum of the patient exposure times. Integument-related toxicities (skin, eye, hair, nail toxicities and cheilitis) were AEs of particular interest in this study. The maximum on-study integument-related toxicity was reported by severity grade. Kaplan-Meier estimates of time to and duration of first integument-related toxicity were prepared. Tolerability was assessed overall in the safety analysis set (all patients who provided informed consent, were enrolled, and who received ≥1 dose of panitumumab), and the *KRAS* safety analysis set (as above but patients also had evaluable *KRAS* status data [i.e. identical to the primary analysis set]).

An analysis of best overall response (assessed using modified Response Evaluation Criteria in Solid Tumours [19]) by grade of skin toxicity (none/mild [grade 0/1] or moderate/severe [grade 3/4]) was also performed.

### Healthcare resource utilisation

Healthcare resource utilisation during the study, including reason for admission and duration of stay in hospital were reported.

### PRO analyses

Quality of life was measured using two validated PRO tools, the EuroQoL EQ-5D [20] and the EORTC quality of life Questionnaires (QLQ-C30) [21]. The EuroQoL EQ-5D is a 5-item scale assessing mobility, self care, usual activities, pain/discomfort and anxiety/depression to produce a single value, the EQ-5D health state index score. Health state index scores range from -0.594 to 1.0 (higher scores represent better health [1.0 = perfect health]). This questionnaire also contains a 0 to 100 visual analogue scale that generates the EQ-5D overall health rating score. The minimally important differences for the health state index and overall health rating scores are considered to be 0.08 and 7, respectively [22]. The EORTC QLQ-C30 includes five functional scales (physical, role, cognitive, emotional and social), three symptom subscales (fatigue, pain and nausea/vomiting), a global health status/quality of life scale, several single items assessing additional symptoms (dyspnoea, insomnia, appetite loss, constipation and diarrhoea) and an item assessing perceived financial impact. Global health status scores range from 0 to 100 (higher scores represent better health).

PRO were an exploratory endpoint and so all analyses are descriptive. PRO analyses were performed on the *KRAS* PRO analysis sets; data were summarised overall and by *KRAS* mutation status. The *KRAS* PRO analysis set comprised enrolled patients who provided informed consent, received ≥1 dose of panitumumab, had evaluable *KRAS* data, and had baseline and ≥1 post-baseline assessment of EQ-5D or QLQ-C30, as appropriate. The PRO analysis set also included patients with unevaluable *KRAS* status. Mean (standard deviation [SD]) baseline, safety follow-up and change from baseline to safety follow-up scores were calculated using actual values. An analysis of change from baseline EQ-5D health state index score by best overall response was also performed. The impact of skin toxicity grade (0/1 or 3/4) on changes from baseline in health state index, overall health rating and global health status scores was estimated using a linear mixed effects model for repeated measures. Least squares means estimates of average change from baseline within each group and the difference between the two groups, along with 95% CIs were calculated in the absence of significant interaction terms in the model.

## Results

### Patients and treatment

Overall, 154 patients were enrolled between May 2007 and June 2008; 137 were included in the PRO analysis set (WT *KRAS n* = 76, MT *KRAS n* = 54, *KRAS* unevaluable *n* = 7). Seventeen patients lacking either baseline or post-baseline PRO assessments were excluded from the PRO analysis set. Overall, the mean (SD) follow-up time was 37.7 (15.7) weeks (WT *vs.* MT *KRAS*: 39.5 weeks *vs.* 35.8 weeks); eight patients were still receiving at least one component of treatment at the time of analysis. Most characteristics were similar between *KRAS* groups, although the MT *KRAS* PRO population included proportionally more women (20% *vs.* 46%), elderly patients (≥75 years of age; 7% *vs.* 19%) and patients with colon cancer (57% *vs.* 67%) compared with the WT population (Table 1). Overall, more men were included in this study than women (69% *vs.* 31%).

**Table 1:** **Patient demographics and diseasecharacteristics at baseline**

	***KRAS *PRO analysis set**		**PRO analysis set**
	**WT *KRAS *(*n*** **= 76)**	**MT *KRAS *(*n*** **= 54)**	**Overall (*n*** **= 137)**
**Sex, *n *(%)**			
Male	61 (80)	29 (54)	95 (69)
Female	15 (20)	25 (46)	42 (31)
**Race/ethnicity, *n *(%)**			
White/Caucasian	73 (96)	53 (98)	133 (97)
Black/African American	2 (3)	0 (0)	2 (1)
Hispanic/Latino	0 (0)	1 (2)	1 (1)
Japanese	1 (1)	0 (0)	1 (1)
**Median age, years (range)**	63.5 (21–84)	65.0 (37–80)	64.0 (21–84)
**Age group, *n *(%)**			
<65 years	40 (53)	26 (48)	71 (52)
≥65 years	36 (47)	28 (52)	66 (48)
<75 years	71 (93)	44 (81)	121 (88)
≥75 years	5 (7)	10 (19)	16 (12)
**Primary tumour type, *n *(%)**			
Colon cancer	43 (57)	36 (67)	82 (60)
Rectal cancer	33 (43)	18 (33)	55 (40)
**Number of metastatic sites, *n *(%)**			
1	33 (43)	23 (43)	63 (46)
2	22 (29)	19 (35)	41 (30)
≥3	21 (28)	12 (22)	33 (24)
**Location of metastatic sites, *n *(%)**			
Liver only	28 (37)	15 (28)	46 (34)
Liver plus other sites	35 (46)	25 (46)	60 (44)
Other sites only	13 (17)	14 (26)	31 (23)
**ECOG performance status, *n *(%)**			
0	43 (57)	30 (56)	80 (58)
1	30 (39)	20 (37)	50 (36)
2	3 (4)	4 (7)	7 (5)
**Mean (SD) PRO scores**			
EQ-5D health state index	0.81 (0.22)	0.80 (0.22)	0.81 (0.22)
EQ-5D overall health rating	71.7 (20.1)	71.9 (19.6)	72.5 (19.8)
QLQ-C30 global health status	64.4 (22.5)	65.1 (22.8)	65.8 (22.5)

*ECOG* Eastern Oncology Group; *PRO* patient-reported outcome.

In the *KRAS* safety analysis set the mean (SD) cumulative panitumumab dose (77.0 [43.2] mg/kg *vs.* 62.2 [34.6] mg/kg) and number of panitumumab cycles delivered (13.2 [7.5] *vs.* 10.9 [6.1]) were higher in the WT *vs.* the MT *KRAS* group. The mean cumulative chemotherapy dose delivered (any component) was also higher in the WT *KRAS* group.

### Integument-related toxicity

Nearly all patients (98%) experienced integument-related toxicities; these comprised skin (97%), eye (38%), hair (38%) and nail (32%) toxicities, and cheilitis (3%). More patients in the WT *KRAS* group had eye toxicities compared with the MT group (45% *vs.* 29%) whereas more patients with MT *KRAS* tumours experienced hair (31% *vs.* 51%) and nail (29% *vs.* 37%) toxicities. Differences in exposure-adjusted AE rates between the WT and MT *KRAS* groups were observed for integument-related toxicities (2938.8 *vs.* 3284.4 events per 100 patient-years, respectively). Grade 3 or higher integument-related toxicity rates were 68.8 and 106.5 events per 100 patient-years (WT *vs.* MT *KRAS* groups, respectively).

Overall, the most frequently reported integument-related AEs were rash (42%), dry skin (40%), acne (36%), alopecia (34%), paronychia (25%), conjunctivitis (21%), and dermatitis acneiform (21%) (Table 2). Rash (43% *vs.* 26%), dry skin (45% *vs.* 36%), conjunctivitis (27% *vs.* 14%), skin fissures (23% *vs.* 17%), pruritus (24% *vs.* 14%), skin toxicity (16% *vs.* 7%), and erythema (12% *vs.* 7%) were more common in the WT *KRAS* group. Alopecia (29% *vs.* 44%) and palmar-plantar erythrodysesthesia (14% *vs.* 22%) were more common in the MT group. Overall, 45% of integument-related toxicities were grade 2 in severity; 36% of patients experienced grade 3 or higher toxicities. Grade 3 or higher toxicities reported in >5 patients included rash, acne and paronychia. In general, there was no difference between *KRAS* groups in the incidence of grade 3 or higher AEs, with the exception of acne (7% *vs.* 14%) and palmar-plantar erythrodysesthesia syndrome (1% *vs.* 3%), which were reported more frequently in the MT *KRAS* group.

**Table 2:** **Most common integument-related toxicities(any grade; incidence ≥10%in the overall group)**

	***n *(%)**					
	**WT *KRAS *(*n*** **= 86)**		**MT *KRAS *(*n*** **= 59)**		**Overall (*n*** **= 154)**	
	**Any grade**	**Grade ≥3**	**Any grade**	**Grade ≥3**	**Any grade**	**Grade ≥3**
Any integument-related AE	84 (98)	29 (34)	58 (98)	22 (37)	151 (98)	55 (36)
Rash	37 (43)	7 (8)	21 (26)	6 (10)	64 (42)	15 (10)
Dry skin	39 (45)	0 (0)	21 (36)	1 (2)	61 (40)	1 (1)
Acne	29 (34)	6 (7)	22 (37)	8 (14)	55 (36)	15 (10)
Alopecia	25 (29)	1 (1)	26 (44)	2 (3)	52 (34)	3 (2)
Paronychia	20 (23)	6 (7)	15 (25)	2 (3)	38 (25)	10 (6)
Conjunctivitis	23 (27)	1 (1)	8 (14)	1 (2)	33 (21)	3 (2)
Dermatitis acneiform	19 (22)	5 (6)	13 (22)	3 (5)	33 (21)	8 (5)
Skin fissures	20 (23)	3 (3)	10 (17)	1 (2)	32 (21)	4 (3)
Pruritus	21 (24)	1 (1)	8 (14)	0 (0)	30 (19)	1 (1)
PPE syndrome	12 (14)	1 (1)	13 (22)	2 (3)	25 (16)	3 (2)
Skin toxicity	14 (16)	4 (5)	4 (7)	1 (2)	18 (12)	5 (3)
Erythema	10 (12)	2 (2)	4 (7)	1 (2)	16 (10)	3 (2)

*AE* adverse event; *PPE* palmar-plantar erythrodysaesthesia.

Overall, the median time to first integument-related toxicity was 8 days (WT *vs.* MT *KRAS* group: 8 days *vs.* 10 days (Table 3)) and the median duration of integument-related toxicity was 334 days (WT *vs.* MT *KRAS* group: 452 *vs.* 321 days). Overall, the median time from last panitumumab dose to resolution of integument-related toxicity was 71 days (WT *vs.* MT *KRAS* group: 103 *vs.* 71 days).

**Table 3:** **Kaplan-Meier analysis of integument-relatedtoxicities**

	**Median (95% CI)**		
	**WT *KRAS***	**MT *KRAS***	**Overall**
	**(*n*** **= 86)**	**(*n*** **= 59)**	**(*n*** **= 154)**
**Time to first integumenttoxicity, days [min, max]**			
Any grade	8.0 (7,0, 10.0)	10.0 (7.0, 13.0)	8.0 (7.0, 11.0)
	[0, 155]	[0, 125]	[0, 155]
Grade ≥3	NE (300.0, NE)	NE (175.0, NE)	408.0 (300.0, NE)
	[6, 528]	[0, 389]	[0, 528]
**Duration of integument toxicity,days [min, max]**			
Any grade	452.0 (244.0, NE)	321.0 (228.0, 371.0)	334.0 (244.0, NE)
	[14, 492]	[19, 445]	[14, 492]
Grade ≥3	32.0 (17.0, 43.0)	55.5 (25.0, 80.0)	36.0 (25.0, 54.0)
	[5, 322]	[5, 268]	[5, 322]
**Time to resolution, days[min, max]**			
Any grade	103.0 (55.0, NE)	71.0 (52.0, 86.0)	71.0 (59.0, 162.0)
	[0, 309]	[5, 162]	[0, 309]
Grade ≥3	103.0 (68.0, NE)	86.0 (86.0, 162.0)	86.0 (68.0, 162.0)
	[0, 124]	[12, 162]	[0, 162]

*NE* not estimable.

#### Response by skin toxicity grade

Of the 152 patients (49%) with measurable disease, 75 (49%) had an objective (complete or partial) response (48/85 [56%] patients *vs.* 22/58 [38%] patients in the WT *vs.* MT *KRAS* groups). Overall, objective responses occurred more commonly in patients with grade 2+ skin toxicity (56%) than in those with grade 0/1 toxicity (29%) (Table 4). Similar observations were noted in the WT and MT *KRAS* groups.

**Table 4:** **Objective response rate byworst skin toxicity grade**

**Objective response rate, %(95% CI)**								
**WT *KRAS***			**MT *KRAS***			**Overall**^**a**^		
**Grade 0/1(*n*** **= 16)**	**Grade2+ (*n*** **= 69)**	**Overall(*n*** **= 85)**	**Grade0/1 (*n*** **= 19)**	**Grade2+ (*n*** **= 39)**	**Overall(*n*** **= 58)**	**Grade0/1 (*n*** **= 35)**	**Grade2+ (*n*** **= 117)**	**Overall(*n*** **= 152)**
37.5 (15.2, 64.6)	60.9 (48.4, 72.4)	56.5 (45.3, 67.2)	21.0 (6.0, 45.6)	46.2 (30.1, 62.8)	37.9 (25.5, 51.6)	28.6 (14,6, 46.3)	55.6 (46.1, 64.7)	49.3 (41.2, 57.6)

Objective response rate represents the proportion of patients with a best overall response of complete or partial response.

^a^All patients in the tumour response analysis set regardless of *KRAS* evaluability.

However, these data may be confounded by the higher mean panitumumab exposure seen in responding *vs.* non-responding patients, overall and in the *KRAS* WT and MT groups (Table 5).

**Table 5:** **Treatment exposure in respondersand non-responders**

	**WT *KRAS *(*n*** **= 85)**		**MT *KRAS *(*n*** **= 58)**		**Overall**^**a**^**(*n*** **= 152)**	
	**Responders (*n*** **= 48)**	**Non-responders (*n*** **= 37)**	**Responders (*n*** **= 22)**	**Non-responders (*n*** **= 36)**	**Responders (*n*** **= 75)**	**Non-responders (*n*** **= 77)**
Panitumumab cycles delivered – mean (SD)	16.3 (6.6)	9.2 (6.9)	15.0 (6.4)	8.5 (4.4)	15.8 (6.6)	8.7 (5.7)
Cumulative panitumumab dose delivered^b^, mg/kg - mean (SD)	94.2 (37.0)	55.2 (41.5)	83.5 (37.4)	49.8 (26.2)	89.8 (37.5)	51.4 (34.2)
Irinotecan cycles delivered mean (SD)	15.6 (6.0)	9.5 (6.3)	15.7 (4.8)	9.3 (4.5)	15.5 (5.7)	9.4 (5.4)
Cumulative irinotecan dose delivered^b^, mg/kg - mean (SD)	2635 (1012)	1614 (1026)	2603 (754)	1542 (722)	2605 (954)	1569 (869)

Responders comprise those patients with a best overall response of a complete or partial response; non-responders comprise those patients with a best overall response of stable disease, disease progression or those with no post-baseline response assessment.

^a^All patients in the tumour response analysis set regardless of *KRAS* evaluability; ^b^Adjusted for weight or BSA (most recent measurement before infusion).

### Healthcare resource utilisation

Overall, 64% of patients were hospitalised during the study; the most common reasons for hospitalisation were chemotherapy (61%), AEs (24%) and normal clinical practice (9%). The median duration of hospital stay was 3 days. More patients with WT *KRAS* status were hospitalised compared with those with MT *KRAS* (70% *vs.* 56%); reasons for hospitalisation were similar between groups and the median duration of hospital stay was the same.

### PRO analyses

#### EuroQoL EQ-5D health state index and overall health rating

**Figure 1:**
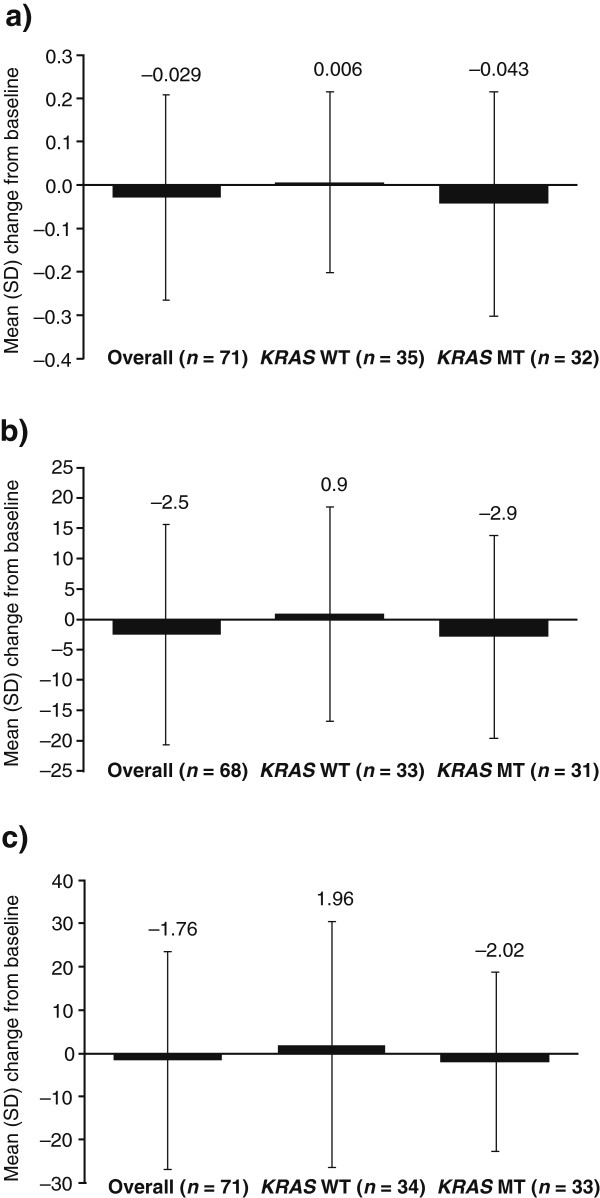
**Change in EQ-5D a)health state index b)overall health rating c)QLQ-C30 global health status.** Data are presented as mean (SD) change in score from baseline to safety follow-up.

Mean overall EQ-5D health state index and health rating scores remained stable throughout the study; baseline and safety follow-up scores for these two scales were also similar in the WT and MT *KRAS* groups (Figure 1).

There appeared to be a trend for better tumour response in patients with higher baseline health state index scores in both the WT and MT *KRAS* groups. Mean (SE) baseline scores for responding patients *vs.* progressing patients with WT *KRAS* tumours were 0.81 (0.03) *vs.* 0.72 (0.18), respectively; mean scores in responding *vs.* progressing patients with MT *KRAS* tumours were 0.82 (0.05) *vs.* 0.59 (0.20). Mean (SE) change from baseline in health state index score in responding patients with WT *KRAS* tumours were positive throughout the study: +0.05 (0.03), +0.07 (0.03), +0.05 (0.03), +0.02 (0.03), +0.05 (0.04) at weeks 8, 16, 24, 32 and safety follow-up visit, respectively. No consistent trends were observed in any other subgroup.

#### EuroQoL EQ-5D subscales

Overall, mean mobility, self care, usual activities, pain/discomfort and anxiety/depression subscale scores remained stable between baseline and safety follow-up (Table 6). Results were generally similar between WT and MT *KRAS* groups, however, there was high within-group variability in scores.

**Table 6:** **PRO EQ-5D and QLQ-C30subscale scores at baselineand safety follow-up**

	***KRAS *PRO analysis set**				**PRO analysis set**	
	**WT *KRAS *(*n*** **= 76)**^**a**^		**MT *KRAS *(*n*** **= 54)**^**a**^		**Overall (*n*** **= 137)**^**a**^	
	**Baseline**	**Safety follow-up**	**Baseline**	**Safety follow-up**	**Baseline**	**Safety follow-up**
**Mean (SD) EQ-5D subscalescores**^**b**^						
Mobility	1.13 (0.34)	1.17 (0.38)	1.13 (0.34)	1.12 (0.42)	1.12 (0.33)	1.16 (0.41)
Self care	1.05 (0.23)	1.14 (0.35)	1.13 (0.44)	1.15 (0.51)	1.08 (0.32)	1.15 (0.43)
Usual activities	1.29 (0.56)	1.39 (0.60)	1.35 (0.59)	1.34 (0.65)	1.30 (0.56)	1.38 (0.62)
Pain/discomfort	1.45 (0.58)	1.53 (0.56)	1.53 (0.58)	1.46 (0.56)	1.46 (0.57)	1.51 (0.56)
Anxiety/depression	1.26 (0.50)	1.25 (0.44)	1.39 (0.49)	1.33 (0.48)	1.31 (0.49)	1.30 (0.46)
**Mean (SD) QLQ-C30 functioningscores**^**c**^						
Physical	71.7 (20.1)	71.3 (20.5)^d^	71.9 (19.6)	78.6 (10.5)^d^	72.5 (19.8)	74.3 (18.2)^d^
Role	26.1 (33.4)	30.0 (33.3)	32.1 (35.0)	28.8 (29.8)	27.4 (33.7)	30.3 (31.2)
Cognitive	10.2 (17.7)	13.3 (19.7)	13.6 (22.7)	14.1 (18.2)	11.0 (19.6)	14.4 (19.6)
Emotional	24.3 (23.1)	22.1 (20.0)	26.5 (22.9)	14.9 (19.0)	24.6 (22.6)	19.8 (20.5)
Social	25.8 (33.4)	25.7 (27.8)	23.5 (32.6)	16.7 (21.2)	23.6 (32.5)	22.4 (24.9)
**Mean (SD) QLQ-C30 symptomscores**^**e**^						
Fatigue	29.0 (27.8)	31.9 (28.3)	35.4 (31.6)	31.3 (22.6)	31.4 (28.8)	32.3 (26.0)
Pain	19.1 (27.4)	22.9 (27.4)	25.3 (29.6)	19.3 (26.1)	21.2 (27.9)	21.6 (26.6)
Nausea/vomiting	3.3 (10.6)	7.6 (14.2)	8.6 (19.1)	10.6 (19.9)	5.3 (14.6)	8.6 (16.8)
**Mean (SD) QLQ-C30 single-itemscores**^**e**^						
Dyspnoea	17.1 (27.5)	21.0 (28.1)	23.5 (30.8)	21.2 (21.8)	19.2 (28.5)	21.3 (26.4)
Insomnia	29.8 (35.8)	23.8 (28.7)	28.4 (32.0)	23.2 (27.0)	29.4 (34.2)	25.0 (28.9)
Appetite loss	18.9 (31.0)	15.2 (30.6)	25.9 (35.3)	15.2 (27.8)	20.7 (32.4)	14.4 (28.4)
Constipation	13.3 (23.2)	4.8 (14.3)	14.2 (25.6)	12.1 (23.3)	14.0 (23.8)	9.7 (22.0)
Diarrhoea	20.7 (30.6)	12.4 (23.0)	19.1 (30.8)	22.2 (27.2)	19.5 (30.0)	18.1 (25.6)
Financial impact	8.1 (20.5)	7.6 (18.2)	6.8 (17.6)	11.5 (26.2)	7.2 (18.8)	9.4 (22.0)

^a^Actual ‘*n*’ number may differ according to subscale item and time point (data from fewer patients were available at safety follow-up *vs.* baseline); ^b^Higher scores indicate a better level of functioning of a greater degree of symptomology depending on subscale; ^c^A higher score indicates a better level of functioning/health; ^c^A higher score indicates a better level of functioning/health; ^d^Data not available at safety follow-up, data reported at week 48 instead; ^e^A higher score indicates a greater degree of symptomology.

#### EORTC QLQ-C30 global health status

Overall, mean global health status scores remained stable throughout the study; data were similar in the WT and MT *KRAS* groups (Figure 1).

### EORTC QLQ-C30 functioning and symptoms subscales and single-items

Overall, no clinically meaningful changes in mean functioning and symptoms scores occurred during the study either overall or by *KRAS* group (Table 6). Similarly, mean single-item scores showed little change and results were similar in each *KRAS* group (Table 5).

### PRO by skin toxicity grade

Based on the repeated measures model, the differences in change from baseline EQ-5D health state index and overall health rating scores between the two skin toxicity groups (grade 0/1 or 2+) were not statistically significant (Table 7). For QLQ-C30 global health status, the model yielded a significant difference between these two groups but there were also interactions between baseline global health status score and skin toxicity overall in the PRO analysis set and within the MT *KRAS* PRO population (data not shown).

**Table 7:** **Change in EQ-5D healthstate index and EQ-5Doverall health rating byseverity of skin toxicity**

	**Least-squares adjusted**^**a**^**mean (95% CI)**		
	**WT *KRAS *(*n*** **= 76)**^**b**^	**MT *KRAS *(*n*** **= 54)**^**b**^	**Overall (*n*** **= 137)**^**b**^
**EQ-5D health state index**			
Worst ST grade 0/1	0.03 (-0.10, 0.15)	0.01(-0.12, 0.14)	0.01 (-0.08, 0.09)
Worst ST grade 2+	0.04 (-0.01, 0.09)	-0.02 (-0.11, 0.06)	0.00 (-0.05, 0.04)
Difference	-0.01 (-0.14, 0.12)	0.03 (-0.12, 0.19)	0.01 (-0.09, 0.10)
**EQ-5D overall health rating**			
Worst ST grade 0/1	-2.4 (-19.1, 14.4)	-3.5 (-12.2, 5.2)	-3.4 (-10.9, 4.2)
Worst ST grade 2+	4.2 (-2.5, 10.9)	-6.1 (-12.0,-0.2)	-1.8 (-5.9, 2.2)
Difference	-6.6 (-24.6, 11.5)	2.6 (-8.0, 13.2)	-1.5 (-10.1, 7.1)

Data are presented as least-squares adjusted mean (95% CI) change in score from baseline through disease progression.

^a^Mixed effect model including fixed effects of time, skin toxicity, baseline EQ-5D score, and the interaction between time and skin toxicity (ST). Interaction of ST with baseline EQ-5D score with *P* < 0.1 was also included in the final model, as were random effects for intercept and time; ^b^Total ^n^ shown; actual *n* number varies for each ST subgroup and scale.

## Discussion

Panitumumab plus FOLFIRI demonstrated acceptable tolerability in this study, with a profile similar to that seen for this combination in the second-line setting [4]. The type and incidence of integument-related toxicities was consistent with that expected for an anti-EGFR antibody plus FOLFIRI and was similar to that observed with cetuximab when administered with irinotecan-based chemotherapy in this setting [3]. The median duration of integument-related toxicity and time taken for resolution of such toxicity was longer in the WT *KRAS* group, perhaps reflecting the higher mean cumulative panitumumab dose and number of panitumumab cycles delivered in this group compared with the MT group. Interestingly, the exposure-adjusted AE rates showed that integument-related toxicities (both overall and ≥ grade 3) were higher in the MT *vs.* the WT *KRAS* group. However, any apparent differences between *KRAS* groups should be interpreted within the context of the relatively small sample size included in this study.

Overall, patients with grade 2+ *vs.* grade 0/1 skin toxicity appeared more likely to respond to treatment, irrespective of tumour *KRAS* status, even though patients with MT *KRAS* tumours are not thought to benefit from panitumumab. However, as noted above, data from this study are potentially confounded by small patient numbers and differences in treatment exposure. For example, responding patients are likely to undergo a longer duration of treatment, which in turn is likely to lead to greater cumulative toxicity. Nonetheless, an association between severity of skin toxicity and improved outcome has previously been noted for both cetuximab [23-25] and panitumumab [26-29] in large phase III trials. The observation in the present study that patients with MT *KRAS* tumours also had better outcomes when they experienced grade 2+ *vs.* grade 0/1 skin toxicity, is in line with other reports suggesting that EGFR-related skin toxicity may be a prognostic rather than predictive marker of outcome during therapy [25,27]. For example, in an analysis of efficacy by skin toxicity grade from the PRIME study, significantly improved progression-free survival (PFS) and OS outcomes were observed in those experiencing higher grades of skin toxicity during panitumumab plus FOLFOX4 treatment, irrespective of tumour *KRAS* status [27]. Furthermore, in the recent German AIO CRC 0104 study, although overall, numeric differences in objective response rate, PFS and OS were observed between patients experiencing grade 2–3 *vs.* 0*–*1 skin toxicity during cetuximab plus chemotherapy treatment, these differences became statistically significant when the group of patients with tumours harbouring codon 12-mutated tumours were examined [25]. In subsequent multivariate analyses, male gender and younger age were significantly correlated with skin toxicity, but no correlation was found with molecular parameters including *KRAS* mutation, EGFR status (by fluorescence *in situ* hybridisation or immunohistochemistry) and EGFR intron-1 polymorphism status [25]. These data suggest that the reported association between EGFR inhibitor-related skin toxicity grade and outcome more likely relates to other factors in the patient impacting on prognosis than alterations in the EGFR pathway in the tumour, but further studies are required to more fully investigate the potential prognostic implications.

EGFR inhibitor-induced rash can have a negative impact on quality of life [30] and proactive management is recommended [31]. A recent study demonstrated that pre-emptive treatment is well tolerated and that patients receiving such treatment during panitumumab therapy had fewer grade 2+ skin toxicities and less quality of life impairment than those assigned to reactive treatment after skin toxicity had developed [32]. Interestingly, not all studies have reported a negative impact of skin toxicity and/or anti-EGFR treatment on quality of life. In a study comparing panitumumab monotherapy plus best supportive care with best supportive care alone, more bothersome skin toxicity according to the modified dermatology life quality index (mDLQI) was associated with improved CRC symptoms and quality of life, and longer PFS and OS in panitumumab-treated patients [26]. Quality of life was also maintained or deteriorations lessened for cetuximab plus best supportive care *vs.* best supportive care alone in another study [33]. Further trials have shown either improvements or no deleterious impact on quality of life when cetuximab was given alongside irinotecan [34] or FOLFIRI [35]*vs.* irinotecan/FOLFIRI alone. Results from the present study are generally in line with these observations; panitumumab plus FOLFIRI had minimal impact on quality of life as EQ-5D and QLQ-C30 scores remained stable throughout the study, despite the high incidence of integument-related toxicity. In line with this, in these present analyses, skin toxicity grade did not appear to significantly affect the overall change in quality of life during the study. To our knowledge, this is the first published report in which the impact of skin toxicity grade on quality or life (EQ-5D health state index and overall health rating scales) was directly assessed, and therefore adds to previous reports suggesting that EGFR-targeted mAb therapy in general or level of skin toxicity bother do not adversely impact on a patient's quality of life.

It appears that baseline EQ-5D scores may be prognostic of best tumour response in patients receiving panitumumab plus FOLFIRI. Overall, however, the PRO data showed a high degree of within-group variability due to the relatively small patient numbers in each subgroup and so any apparent differences should be interpreted with caution. Nonetheless, higher baseline quality of life has previously been associated with improved PFS and/or OS in patients with advanced gastrointestinal/colorectal tumours [36,37]. Although to our knowledge, this is the first report of improved quality of life specifically in patients responding to panitumumab plus FOLFIRI treatment, it is perhaps not unexpected as similar observations have been reported previously in patients with advanced gastrointestinal/colorectal tumours undergoing treatment with chemotherapy alone [38,39]. These observations are also in line with the well-documented association between baseline performance status and OS in colorectal cancer [40].

## Conclusions

First-line panitumumab plus FOLFIRI treatment has an acceptable tolerability profile, in line with that expected for such a combination in this setting. Furthermore, treatment appeared to have little impact on quality of life, despite the high incidence of integument-related toxicity. However, the relatively small patient number in each subgroup gave rise to large variations in scores on the various PRO scales, and no clear differences emerged between WT and MT *KRAS* groups in these exploratory analyses. These results provide valuable additional insights into the integument-related toxicities associated with panitumumab, the time taken to develop such toxicities and their likely duration, as well as adding to the body of evidence evaluating the impact of skin toxicity severity on treatment efficacy and patient quality of life. Such insights could help improve patient management; by providing healthcare providers with the most up-to-date information regarding these toxicities they are able to better inform their patients and reassure them about the likely lack of impact on their overall quality of life.

## Competing interests

JT and RH have received honoraria and research funding from Amgen Ltd; MK has received honoraria from Amgen Ltd and has also acted as a consultant to Amgen Ltd; HL has acted as a consultant/advisor to Amgen Ltd and Roche Ltd; CHK has acted as a consultant/advisor to Amgen Ltd, Roche Ltd and Merck KG Damstadt; AB and EG are employees of Amgen Ltd; LM, RG and EF have no competing interests to declare.

## Author' contributions

JT, MK, LM, RG, HL, RH, EF and CHK all made substantial contributions to the acquisition and interpretation of data and were involved in drafting and/or critically revising the manuscript for important intellectual content. EG made substantial contributions to the design of the study and critically revised the manuscript for important intellectual content. AB made substantial contributions to the data analysis and critically revised the manuscript for important intellectual content. All authors approved the final version of this paper ahead of publication.

## Pre-publication history

The pre-publication history for this paper can be accessed here:

http://www.biomedcentral.com/1471-2407/12/438/prepub

## References

[B1] National Comprehensive Cancer Network® NCCN Colon Cancer Guidelines™ Version 1 2011 http://www.nccn.org

[B2] Sobrero A Ackland S Clarke S Perez-Carrion R Chiara S Gapski J Mainwaring P Langer B Young S Phase IV study of bevacizumab in combination with infusional fluorouracil, leucovorin and irinotecan (FOLFIRI) in first-line metastatic colorectal cancer Oncology 2009 77 113 119 19628950 10.1159/000229787

[B3] Van Cutsem E Köhne CH Hitre E Zaluski J Chang Chien CR Makhson A D'Haens G Pinter T Lim R Bodoky G Roh JK Folprecht G Ruff P Stroh C Tejpar S Schlichting M Nippgen J Rougier P Cetuximab and chemotherapy as initial treatment for metastatic colorectal cancer N Engl J Med 2009 360 1408 1417 19339720 10.1056/NEJMoa0805019

[B4] Peeters M Price TJ Cervantes A Sobrero AF Ducreux M Hotko Y Andre T Chan E Lordick F Punt CJ Strickland AH Wilson G Ciuleanu TE Roman L Van Cutsem E Tzekova V Collins S Oliner KS Rong A Gansert J Randomized phase III study of panitumumab with fluorouracil, leucovorin, and irinotecan (FOLFIRI) compared with FOLFIRI alone as second-line treatment in patients with metastatic colorectal cancer J Clin Oncol 2010 28 4706 4713 20921462 10.1200/JCO.2009.27.6055

[B5] Davies JM Goldberg RM First-line therapeutic strategies in metastatic colorectal cancer Oncology 2008 22 1470 1479 19133603

[B6] Grenon NN Chan J Managing toxicities associated with colorectal cancer chemotherapy and targeted therapy: a new guide for nurses Clin J Oncol Nurs 2009 13 285 296 19502186 10.1188/09.CJON.285-296

[B7] Douillard JY Sobrero A Carnaghi C Comella P Diaz-Rubio E Santoro A Van Cutsem E Metastatic colorectal cancer: integrating irinotecan into combination and sequential chemotherapy Ann Oncol 2003 14 Suppl 2 ii7 ii12 12810451 10.1093/annonc/mdg723

[B8] Lacouture ME Mechanisms of cutaneous toxicities to EGFR inhibitors Nat Rev Cancer 2006 6 803 812 16990857 10.1038/nrc1970

[B9] Galimont-Collen AF Vos LE Lavrijsen AP Ouwerkerk J Gelderblom H Classification and management of skin, hair, nail and mucosal side-effects of epidermal growth factor receptor (EGFR) inhibitors Eur J Cancer 2007 43 845 851 17289377 10.1016/j.ejca.2006.11.016

[B10] Giusti RM Shastri K Pilaro AM Fuchs C Cordoba-Rodriguez R Koti K Rothmann M Men AY Zhao AH Hughes M Keegan P Weiss KD Pazdur R U.S. Food and Drug Administration approval: panitumumab for epidermal growth factor receptor-expressing metastatic colorectal carcinoma with progression following fluoropyrimidine-, oxaliplatin-, and irinotecan-containing chemotherapy regimens Clin Cancer Res 2008 14 1296 1302 18316547 10.1158/1078-0432.CCR-07-1354

[B11] Sipples R Common side effects of anti-EGFR therapy: acneform rash Semin Oncol Nurs 2006 22 1 Suppl 1 28 34 16616284 10.1016/j.soncn.2006.01.013

[B12] Wagner LI Lacouture ME Dermatologic toxicities associated with EGFR inhibitors: the clinical psychologist's perspective. Impact on health-related quality of life and implications for clinical management of psychological sequelae Oncology 2007 21 11 Suppl 5 34 36 18154217

[B13] Lacouture ME Insights into the pathophysiology and management of dermatologic toxicities to EGFR-targeted therapies in colorectal cancer Cancer Nurs 2007 30 4 Suppl 1 S17 S26 17666987 10.1097/01.NCC.0000281758.85704.9b

[B14] Köhne CH Hofheinz R Mineur L Letocha H Greil R Thaler J Fernebro E Gamelin E DeCosta L Karthaus M First-line panitumumab plus irinotecan/5-fluorouracil/leucovorin treatment in patients with metastatic colorectal cancer J Cancer Res Clin Oncol 2012 138 65 72 21960318 10.1007/s00432-011-1061-6 PMC11824475

[B15] Amado RG Wolf M Peeters M Van Cutsem E Siena S Freeman DJ Juan T Sikorski R Suggs S Radinsky R Patterson SD Chang DD Wild-type KRAS is required for panitumumab efficacy in patients with metastatic colorectal cancer J Clin Oncol 2008 26 1626 1634 18316791 10.1200/JCO.2007.14.7116

[B16] Di Fiore F Blanchard F Charbonnier F Le Pessot F Lamy A Galais MP Bastit L Killian A Sesboue R Tuech JJ Queuniet AM Paillot B Sabourin JC Michot F Michel P Frebourg T Clinical relevance of KRAS mutation detection in metastatic colorectal cancer treated by Cetuximab plus chemotherapy Br J Cancer 2007 96 1166 1169 17375050 10.1038/sj.bjc.6603685 PMC2360149

[B17] National Cancer Institute Cancer Therapy Evaluation Program (CTEP) Common Toxicity Criteria for Adverse Events v3.0 (CTCAE) 2005 http://ctep.cancer.gov/protocolDevelopment/electronic_applicationa/docs/ctcaev3.pdf

[B18] National Cancer Institute Cancer Therapy Evaluation Program (CTEP) Common Terminology Criteria for Adverse Events (CTCAE), Version 3.0, DCTD, NCI, NIH, DHHS 2006 http://ctep.cancer.gov

[B19] Therasse P Arbuck SG Eisenhauer EA Wanders J Kaplan RS Rubinstein L Verweij J Van Glabbeke M van Oosterom AT Christian MC Gwyther SG New guidelines to evaluate the response to treatment in solid tumors. European Organization for Research and Treatment of Cancer, National Cancer Institute of the United States, National Cancer Institute of Canada J Natl Cancer Inst 2000 92 205 216 10655437 10.1093/jnci/92.3.205

[B20] Rabin R de Charro F EQ-5D: a measure of health status from the EuroQol Group Ann Med 2001 33 337 343 11491192 10.3109/07853890109002087

[B21] Cocks K King MT Velikova G Fayers PM Brown JM Quality, interpretation and presentation of European Organisation for Research and Treatment of Cancer quality of life questionnaire core 30 data in randomised controlled trials Eur J Cancer 2008 44 1793 1798 18599286 10.1016/j.ejca.2008.05.008

[B22] Pickard AS Neary MP Cella D Estimation of minimally important differences in EQ-5D and VAS scores in cancer Health Qual Life Outcomes 2007 5 70 18154669 10.1186/1477-7525-5-70 PMC2248572

[B23] Sohn BS Kim TW Lee JL Ryu MH Chang HM Kang YK Park HS Na YS Jang SJ Kim JC Lee JS The role of KRAS mutations in predicting the efficacy of cetuximab-plus-irinotecan therapy in irinotecan-refractory Korean metastatic colorectal cancer patients Oncology 2009 77 224 230 19738388 10.1159/000236046

[B24] Boccia RV Cosgriff TM Headley DL Badarinath S Dakhil SR A phase II trial of FOLFOX6 and cetuximab in the first-line treatment of patients with metastatic colorectal cancer Clin Colorectal Cancer 2010 9 102 107 20378504 10.3816/CCC.2010.n.014

[B25] Stintzing S Kapaun C Laubendery RP Jung A Neumann J Modest DP Giessen C Moosmann N Wollenberg A Kirchner T Heinemann V Prognostic value of cetuximab related skin toxicity in metastatic colorectal cancer (mCRC) patients and its correlation with parameters of the EGFR signal transduction pathway. Results from a randomized trial of the GERMAN AIO CRC Study Group Int J Cancer 2012 Epub ahead of print 10.1002/ijc.27654 22644776

[B26] Peeters M Siena S Van Cutsem E Sobrero A Hendlisz A Cascinu S Kalofonos H Devercelli G Wolf M Amado RG Association of progression-free survival, overall survival, and patient-reported outcomes by skin toxicity and KRAS status in patients receiving panitumumab monotherapy Cancer 2009 115 1544 1554 19189371 10.1002/cncr.24088

[B27] Price TJ Sobrero AF Wilson G Van Cutsem E Aleknaviciene B Zaniboni A Hartmann JT Tian Y Gansert JL Peeters M Randomized, open-label, phase III study of panitumumab (pmab) with FOLFIRI versus FOLFIRI alone as second-line treatment (tx) in patients (pts) with metastatic colorectal cancer (mCRC): Efficacy by skin toxicity (ST) [abstract] J Clin Oncol 2010 28 Suppl 15 3529

[B28] Douillard JY Siena S Tabernero J Burkes RL Barugel ME Humblet Y Cunningham D Xu F Zhao Z Sidhu R Final skin toxicity (ST) and patient-reported outcomes (PRO) results from PRIME: A randomized phase III study of panitumumab (pmab) plus FOLFOX4 (CT) for first-line metastatic colorectal cancer (mCRC) [abstract] J Clin Oncol 2012 30 Suppl 4 531

[B29] Sobrero AF Peeters M Price TJ Hotko Y Cervantes-Ruiperez A Ducreux M André T Chan E Lordick F Tian Y Sidhu R Final results from study 181: Randomized phase III study of FOLFIRI with or without panitumumab (pmab) for the treatment of second-line metastatic colorectal cancer (mCRC) [abstract] J Clin Oncol 2012 30 Suppl 4 38722215753

[B30] Witherspoon JN Wagner L Rademaker A West DP Rosenbaum SE Lacouture ME Correlation of patient characteristics and NCI-Common Terminology Criteria for Adverse Events (CTCAE) v 3.0 grading with dermatology-related quality of life (QoL) in patients with EGFR inhibitor-induced rash [abstract] J Clin Oncol 2008 26 Suppl 9559

[B31] Melosky B Burkes R Rayson D Alcindor T Shear N Lacouture M Management of skin rash during EGFR-targeted monoclonal antibody treatment for gastrointestinal malignancies: Canadian recommendations Curr Oncol 2009 16 16 26 19229368 10.3747/co.v16i1.361 PMC2644628

[B32] Lacouture ME Mitchell EP Piperdi B Pillai MV Shearer H Iannotti N Xu F Yassine M Skin toxicity evaluation protocol with panitumumab (STEPP), a phase II, open-label, randomized trial evaluating the impact of a pre-Emptive Skin treatment regimen on skin toxicities and quality of life in patients with metastatic colorectal cancer J Clin Oncol 2010 28 1351 1357 20142600 10.1200/JCO.2008.21.7828

[B33] Au HJ Karapetis CS O'Callaghan CJ Tu D Moore MJ Zalcberg JR Kennecke H Shapiro JD Koski S Pavlakis N Charpentier D Wyld D Jefford M Knight GJ Magoski NM Brundage MD Jonker DJ Health-related quality of life in patients with advanced colorectal cancer treated with cetuximab: overall and KRAS-specific results of the NCIC CTG and AGITG CO.17 Trial J Clin Oncol 2009 27 1822 1828 19273701 10.1200/JCO.2008.19.6048

[B34] Sobrero AF Maurel J Fehrenbacher L Scheithauer W Abubakr YA Lutz MP Vega-Villegas ME Eng C Steinhauer EU Prausova A Lenz HJ Borg C Middleton G Kroning H Luppi G Kisker O Zubel A Langer C Kopit J Burris HA III EPIC: phase III trial of cetuximab plus irinotecan after fluoropyrimidine and oxaliplatin failure in patients with metastatic colorectal cancer J Clin Oncol 2008 26 2311 2319 18390971 10.1200/JCO.2007.13.1193

[B35] Lang I Köhne CH Folprecht G Nowacki MP Cascinu S Shchepotin I Maurel J Cunningham D Zubel A Van Cutsem E Cetuximab plus FOLFIRI in 1st-line treatment of metastatic colorectal cancer: Quality of life (QoL) analysis of patients (pts) with KRAS wild-type (wt) tumours in the CRYSTAL trial [abstract] Eur J Cancer Suppl 2009 7 345

[B36] Maisey NR Norman A Watson M Allen MJ Hill ME Cunningham D Baseline quality of life predicts survival in patients with advanced colorectal cancer Eur J Cancer 2002 38 1351 1357 12091066 10.1016/s0959-8049(02)00098-9

[B37] Chau I Norman AR Cunningham D Waters JS Oates J Ross PJ Multivariate prognostic factor analysis in locally advanced and metastatic esophago-gastric cancer–pooled analysis from three multicenter, randomized, controlled trials using individual patient data J Clin Oncol 2004 22 2395 2403 15197201 10.1200/JCO.2004.08.154

[B38] Hobday TJ Kugler JW Mahoney MR Sargent DJ Sloan JA Fitch TR Krook JE O'Connell MJ Mailliard JA Tirona MT Tschetter LK Cobau CD Goldberg RM Efficacy and quality-of-life data are related in a phase II trial of oral chemotherapy in previously untreated patients with metastatic colorectal carcinoma J Clin Oncol 2002 20 4574 4580 12454115 10.1200/JCO.2002.08.535

[B39] Shin DB Bang SM Park SH Kang HG Jue JI Han SH Lee Y Cho EK Lee JH Correlation of quality of life with tumor response in patients receiving palliative chemotherapy for advanced gastrointestinal tumors Med Oncol 2008 25 81 87 18188719 10.1007/s12032-007-0045-5

[B40] Stillwell AP Ho YH Veitch C Systematic review of prognostic factors related to overall survival in patients with stage IV colorectal cancer and unresectable metastases World J Surg 2011 35 684 692 21181473 10.1007/s00268-010-0891-8

